# HLA tapasin independence: broader peptide repertoire and HIV control

**DOI:** 10.1073/pnas.2013554117

**Published:** 2020-10-23

**Authors:** Arman A. Bashirova, Mathias Viard, Vivek Naranbhai, Alba Grifoni, Wilfredo Garcia-Beltran, Marjan Akdag, Yuko Yuki, Xiaojiang Gao, Colm O’hUigin, Malini Raghavan, Steven Wolinsky, Jay H. Bream, Priya Duggal, Jeremy Martinson, Nelson L. Michael, Gregory D. Kirk, Susan P. Buchbinder, David Haas, James J. Goedert, Steven G. Deeks, Jacques Fellay, Bruce Walker, Philip Goulder, Peter Cresswell, Tim Elliott, Alessandro Sette, Jonathan Carlson, Mary Carrington

**Affiliations:** ^a^Basic Science Program, Frederick National Laboratory for Cancer Research, Frederick, MD 21702;; ^b^Department of Medical Oncology, Dana–Farber Cancer Institute, Boston, MA 02215;; ^c^Division of Vaccine Discovery, La Jolla Institute for Immunology, La Jolla, CA 92037;; ^d^Ragon Institute of Massachusetts General Hospital, Massachusetts Institute of Technology and Harvard University, Cambridge, MA 02139;; ^e^Department of Microbiology and Immunology, University of Michigan Medical School, Ann Arbor, MI 48109;; ^f^Division of Infectious Diseases, The Feinberg School of Medicine, Northwestern University, Chicago, IL 60611;; ^g^Department of Molecular Microbiology and Immunology, Johns Hopkins Bloomberg School of Public Health, Baltimore, MD 21205;; ^h^Department of Epidemiology, Johns Hopkins Bloomberg School of Public Health, Baltimore, MD 21205;; ^i^Department of Infectious Diseases and Microbiology, University of Pittsburgh Graduate School of Public Health, Pittsburgh, PA 15261;; ^j^US Military HIV Research Program, Walter Reed Army Institute of Research, Silver Spring, MD 20910;; ^k^ HIV Research Section, San Francisco Department of Public Health, San Francisco, CA 94102;; ^l^Department of Pharmacology, Vanderbilt University School of Medicine, Nashville, TN 37204;; ^m^Infections and Immunoepidemiology Branch, Division of Cancer Epidemiology and Genetics, National Cancer Institute, National Institutes of Health, Rockville, MD 20850;; ^n^Department of Medicine, University of California, San Francisco, CA 94110;; ^o^School of Life Sciences, École Polytechnique Fédérale de Lausanne, 1015 Lausanne, Switzerland;; ^p^Swiss Institute of Bioinformatics, 1015 Lausanne, Switzerland;; ^q^Department of Paediatrics, University of Oxford, Oxford, OX1 4AJ, United Kingdom;; ^r^Department of Immunobiology, Yale University School of Medicine, New Haven, CT 06520;; ^s^Institute for Life Sciences, University of Southampton, Southampton, SO17 1BJ, United Kingdom;; ^t^Centre for Cancer Immunology, University of Southampton, Southampton SO16 6YD, United Kingdom;; ^u^Department of Medicine, University of California San Diego, La Jolla, CA 92093;; ^v^Immunomics, Microsoft Healthcare NExT, Redmond, WA 98052

**Keywords:** HLA, tapasin, peptide repertoire

## Abstract

Human leukocyte antigen (HLA) class I allotypes vary in their ability to present peptides in the absence of tapasin, an essential component of the peptide loading complex. We quantified tapasin dependence of all allotypes that are common in European and African Americans (*n* = 97), which revealed a broad continuum of values. Ex vivo examination of cytotoxic T cell responses to the entire HIV-1 proteome from infected subjects indicates that tapasin-dependent allotypes present a more limited set of distinct peptides than do tapasin-independent allotypes, data supported by computational predictions. This suggests that variation in tapasin dependence may impact the strength of the immune responses by altering peptide repertoire size. In support of this model, we observed that individuals carrying *HLA class I* genotypes characterized by greater tapasin independence progress more slowly to AIDS and maintain lower viral loads, presumably due to increased breadth of peptide presentation. Thus, tapasin dependence level, like *HLA* zygosity, may serve as a means to restrict or expand breadth of the HLA-I peptide repertoire across humans, ultimately influencing immune responses to pathogens and vaccines.

## Significance

HLA class I molecules bind antigenic peptides and present them on the cell surface to cytotoxic T cells to initiate immune responses. The peptide selection process occurs intracellularly with the aid of a molecule called tapasin. HLA class I molecules are highly variable, which influences their structural characteristics and the level of tapasin involvement in peptide selection. We measured tapasin dependence levels of nearly 100 HLA variants and found that the level of tapasin dependence negatively correlates with the number of peptides that the HLA class I molecule presents to T cells, thereby affecting breadth of the immune response. Analysis of HLA genotypes in HIV cohorts reveals that greater tapasin independence associates with slower disease progression and lower viral load.

The classical HLA class I molecules, HLA-A, HLA-B, and HLA-C, present antigenic peptides to CD8+ T cells, eliciting an adaptive immune response (1). They are generally expressed on the cell surface as a trimeric complex consisting of HLA class I heavy chain, β2-microglobulin, and peptide. The genes encoding heavy chains are highly polymorphic, resulting in extensive diversity of the peptide repertoire, both within individuals and at the population level. Peptide loading of HLA class I molecules takes place primarily in the endoplasmic reticulum within the peptide loading complex (PLC) (2). Tapasin is a critical component of the PLC, which performs its peptide “editing” function by association with peptide-empty HLA class I, stabilizing its structure, and promoting dissociation of low affinity peptides (3–13).

HLA class I allotypes vary in level of cell surface expression in the absence of tapasin. Some allotypes are expressed at very low levels on the surface of tapasin-deficient cells (tapasin-dependent allotypes), while others exhibit normal expression on these cells (tapasin-independent allotypes) (14–17). The exact molecular determinants of tapasin dependence (TD) remain unknown, although amino acids in the peptide binding groove near the peptide C terminus appear to contribute most to this phenomenon. For example, a single amino acid change (D116Y) located in this region distinguishes the highly tapasin-dependent B*44:02 allotype from the tapasin-independent B*44:05 allotype (17, 18). Previous data suggest that TD can be defined by the stability of an HLA class I peptide-free form, where the more stable tapasin-independent allotypes are capable of selecting peptides for presentation on their own, while the less stable tapasin-dependent allotypes require chaperone assistance (6, 8, 14, 19).

Tapasin function can be targeted by viruses as a means of downmodulating HLA class I and evading cytotoxic CD8+ T cell (CTL) responses (20, 21). Similarly, loss of tapasin expression has been observed in various human cancers (22). Thus, tapasin-independent HLA class I allotypes may be advantageous in terms of eliciting CTL responses against virally infected cells or tumor cells when tapasin function has been diminished. Allotype-specific regulation of the peptide repertoire by tapasin may also affect the quality of CTL responses. Here, we quantified the level of TD across all common HLA allotypes present in European and African Americans and tested the functional significance of differential HLA class I TD and its impact on disease.

## Results

### TD of Common HLA Class I Allotypes.

A panel of 97 distinct lentiviral HLA class I expression constructs were generated, in which HLA heavy chains were fused with a FLAG tag at the N terminus and linked to a ZsGreen reporter via a self-cleaving peptide for transduction control. The constructs were transduced individually into the human B cell line .220, which does not express tapasin (23), and into tapasin-reconstituted .220 cells (.220tpn). There are two common, naturally occurring tapasin variants, one containing arginine and the other threonine at position 240 of the mature protein (rs2071888). In order to evaluate the potential influence of this polymorphism on HLA class I expression, each variant was transduced separately into .220 cells with a C-terminal V5 tag. The resulting cell lines stably expressed tapasin at the same level as assessed by intracellular staining.

**Fig. 1.**
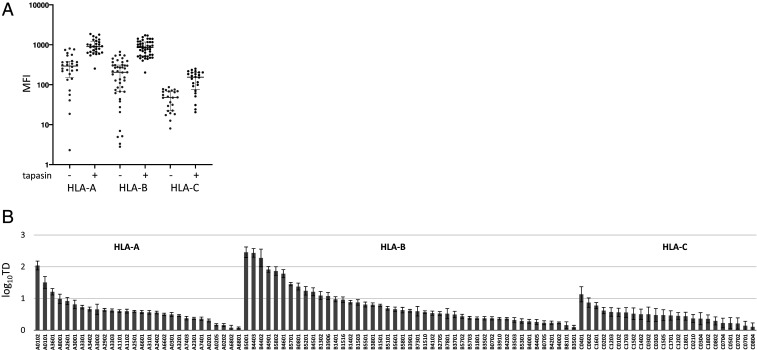
Tapasin influences HLA class I surface expression. (*A*) HLA expression levels in .220 cells before and after tapasin reconstitution were measured by flow cytometry using anti-FLAG mAb. MFI, median fluorescent intensity. Data for tapasin-positive cells represent the average MFI for R240 and T240 tapasin-expressing cells. (*B*) TD for each HLA allotype, defined as the ratio of MFI of tapasin-positive over tapasin-negative cells, is shown in log_10_ scale. Error bars correspond to SDs calculated from multiple measurements as described in *Methods*.

HLA class I cell surface expression levels were quantified in tapasin-deficient and tapasin-sufficient cell lines by flow cytometry using an anti-FLAG mAb (Fig. 1*A* and *SI Appendix*, Fig. S1). This antibody specifically detects transduced allotypes, in contrast to W6/32 mAb, a broadly used pan-HLA class I antibody that can bind endogenous HLA class I, which may be expressed by .220 cells at low levels. Nevertheless, we observed a strong correlation between HLA expression levels measured using anti-FLAG mAb and those determined by staining with W6/32 (*SI Appendix*, Fig. S2). This indicated that the FLAG-tagged heavy chains remained properly folded on the cell surface, since W6/32 only binds β2-microgloblin–associated HLA class I complexes. Further, allotype-specific HLA class I expression levels in .220tpn cells were similar to the expression levels in isogenic .221 cells bearing endogenous tapasin, indicating that V5-tagged tapasin was functioning similarly to the native protein (*SI Appendix*, Figs. S1 *B* and *C* and S3). Comparison of the flow cytometric data obtained from cells expressing the R240 vs. T240 tapasin variants did not reveal a differential effect of this polymorphism on HLA expression levels.

TD values for each allele were generated by calculating the ratio of expression level in .220tpn cells over expression level in .220 cells, such that higher scores for a given allotype indicate its greater dependence on tapasin for cell surface expression (Fig. 1*B* and *SI Appendix*, Table S1). For example, the highly tapasin-dependent HLA-A*01:02 and HLA-B*44:02 have a ratio above 100, whereas the highly tapasin-independent HLA-A*68:01 and HLA-B*35:01 have a ratio of ∼1. TD values across each HLA class I locus represent a continuum, demonstrating some level of dependence on tapasin for most allotypes.

Our dataset included 26 of the 27 *HLA-B* alleles for which expression levels had been determined previously in the tapasin-deficient melanoma cell line M553 using W6/32 for cell surface staining of transfected cells (14). Despite differences in experimental conditions (cell type, DNA delivery, antibody), significant correlations between the .220 and M553 datasets were observed for both expression levels in tapasin-deficient cells and fold increase in expression after tapasin reconstitution (*SI Appendix*, Fig. S4).

### TD Is Associated with Peptide Repertoire Size.

**Fig. 2.**
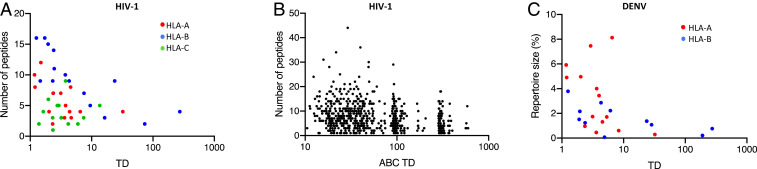
Tapasin dependence levels negatively correlate with the number of peptides presented by HLA allotypes. (*A*) CD8+ T cell responses were measured in an ELISpot assay in a cohort of HIV-1 infected South Africans. The number of peptides eliciting a CD8+ T cell response associated with each HLA allotype (*y* axis) is plotted against the corresponding TD value (*x* axis). Each dot represents an individual HLA allotype. (*B*) The total number of peptides to which an individual elicited responses (*y* axis) is plotted against the global TD value (*x* axis) for that individual. The global score equals the sum of six TD values corresponding to the *HLA-A/B/C* genotype. Each dot represents an individual tested in the ELISpot assay. (*C*) Dengue virus (DENV) repertoire size (*y* axis), as predicted using the SMM algorithm for individual HLA-A and HLA-B allotypes (25), is plotted against the corresponding TD values for HLA-A and HLA-B (*x* axis). Each dot represents a specific HLA allotype.

The kinetics of peptide selection may differ between tapasin-independent allotypes relative to tapasin-dependent allotypes, since peptide selection for tapasin-independent allotypes, which does not require involvement of the PLC, may proceed faster and with somewhat greater promiscuity. If so, TD levels of HLA class I allotypes are likely to influence breadth of the peptide repertoire, i.e., the number of peptides presented by HLA class I on the cell surface. Although multiple factors may influence the immunogenicity of the presented peptide pool, it stands to reason that a greater repertoire of peptides present in the HLA peptide binding groove will generally result in an increased number of immunogenic peptides presented to T cells. Therefore, TD levels may influence the breadth of CD8+ T cell responses by regulating the number of peptides bound to HLA class I. To test this hypothesis, we correlated TD levels with breadth of CD8+ T cell responses against a panel of 410 overlapping synthetic peptides spanning the HIV-1 proteome in a cohort of 989 HIV-1 clade C-infected individuals who were genotyped for *HLA class I* (24). CD8+ T cell responses from each individual were determined by an IFN-γ enzyme-linked immunosorbent (ELISpot) assay, and peptides eliciting a response were tested for association with all *HLA class I* alleles present in the cohort. As a result, each *HLA class I* allele was assigned a certain number of unique HIV-1 peptides that elicited a CD8+ T cell response. Significant negative correlations between the level of TD and the number of peptides associated with each of the HLA-B (*r* = −0.77, *P* = 0.0004), HLA-A (*r* = −0.48, *P* = 0.04), and HLA-A/B/C allotypes combined (*r* = −0.35, *P* = 0.01) were observed (Fig. 2*A* and Table 1). There was no correlation for HLA-C allotypes alone.

**Table 1.** Statistics for Spearman correlation between TD levels and peptide repertoire size corresponding to Fig. 2

		*HLA-A*	*HLA-B*	*HLA-C*	*HLA-A/B*	*HLA-A/B/C*
HIV-1 (allotypes associated CD8+ T cell response) – Fig. 2*A*	*n*	14	16	13	30	43
	*r*	−0.48	−0.77	0.04	−0.57	−0.35
	*P*	**0.04**	**0.0004**	0.5	**0.0005**	**0.01**
HIV-1 (total CD8+ T cell response) – Fig. 2*B*	*n*	638	638	638	638	638
	*r*	−0.05	−0.24	−0.05	−0.26	−0.24
	*P*	0.09	**6 × 10**^**−10**^	0.1	**2 × 10**^**−11**^	**3 × 10**^**−10**^
DENV (predicted repertoire for individual allotypes) – Fig. 2*C*	*n*	14	11		25	
	*r*	−0.46	−0.65		−0.53	
	*P*	**0.05**	**0.02**		**0.003**	

Bold *P* values indicate significance (less than or equal to 0.05).

Next, we tested for associations between TD score of each individual and breadth of their corresponding responses (i.e., number of peptides eliciting a CTL response, regardless of *HLA* genotype). A locus-specific TD score for each individual was calculated as the sum of the TD values for the two alleles at a given locus, or a “global” TD score was generated by summing TD values for the four alleles of their *HLA-A/B* or six alleles of their *HLA-A/B/C* genotypes. The global TD score demonstrated a significant negative correlation with the total number of HIV-1 peptides to which CD8+ T cell responses were detected (*r* = −0.24, *P* = 3 × 10^−10^, Fig. 2*B* and Table 1). As in the analysis of allelic association described above, the *HLA-B* locus was the major contributor to this effect (*r* = −0.24, *P* = 6 × 10^−10^, Table 1).

The correlation between the number of peptides presented and TD level was further examined using data by Paul et al. (25), which demonstrated substantial differences in dengue virus repertoire size for a set of 16 HLA-A and 11 HLA-B allotypes estimated by peptide binding prediction using the stabilized matrix method (SMM) (26). This algorithm is trained on in vitro binding assays, and peptides with predicted affinity (IC_50_) ≤ 500 nM are expected to be presented by the corresponding HLA class I. Similar to the HIV-1 ELISpot data, the predicted dengue repertoire size showed a negative correlation with TD of HLA class I allotypes (Fig. 2*C* and Table 1), which reached significance for HLA-B (*r* = −0.65, *P* = 0.02), HLA-A (*r* = −0.46, *P* = 0.05), and HLA-A/B allotypes combined (*r* = −0.53, *P* = 0.003). The prediction algorithms NetMHCpan EL 4.0 (27) and SMM appear to be robust for viral peptidomes, as significant negative correlations were observed between the predicted number of HIV peptides presented by HLA-A/B (using the same overlapping set of peptides used in the ex vivo analysis in Fig. 2 *A* and *B*) and the corresponding TD scores (*r* = −0.33, *P* = 0.003 and *r* = −0.32, *P* = 0.03, for NetMHCpan EL 4.0 and SMM, respectively). Of note, the order of TD scores of four HLA-B alleles, B*57:01, B*27:05, B*07:02, and B*35:01, corresponds precisely, in a negative manner, to the order of their self-peptide repertoire sizes as estimated using prediction algorithms (28).

Thus, both ELISpot data and computational prediction algorithms indicate that TD of HLA class I allotypes associates with more limited peptide repertoires. The negative correlations are consistent for HLA-A and HLA-B, but not for HLA-C allotypes, an observation that may be due to the relatively low expression of HLA-C in general (29) and greater difficulty in measuring and predicting peptide binding to HLA-C. Notably, the in vitro binding assays used to build the prediction algorithms do not involve tapasin. It is possible that tapasin-independent allotypes bind larger numbers of peptides in these assays due to their higher stability in a peptide-free form, a characteristic that, in the ER, liberates their need for tapasin in the peptide loading process. This may explain the parallel correlations obtained by both ex vivo and prediction methods, attesting to the validity of the latter.

### TD Associates with HIV Disease Outcomes.

**Fig. 3.**
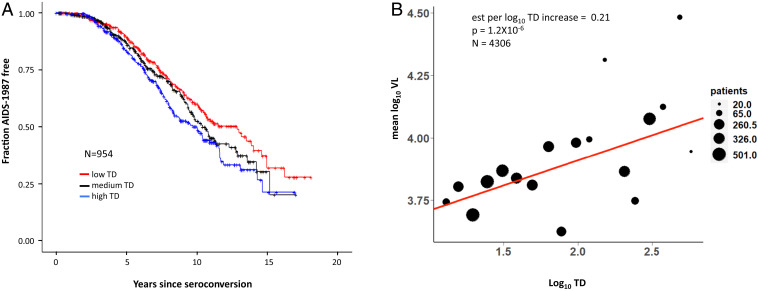
Tapasin dependence impacts HIV-1 disease. (*A*) Kaplan–Meyer curves for time to AIDS-1987 are shown for a cohort of ART-naïve HIV-1 seroconverters equally divided based on their global TD (high, medium, and low, *n* = 318 in each group). (*B*) Mean log_10_VL plotted against log_10_TD level is shown for a cohort of ART-naïve HIV-1 patients. Each dot represents the mean log_10_VL of patient groups divided into increasing bins of 0.1 log_10_TD. Estimate (est), and *P* value were derived by regression analysis adjusted by race.

The expansion or restriction of the peptide binding repertoire as a function of TD level may influence disease pathogenesis. To test this, we assigned TD scores to individuals from two completely independent HIV-1 cohorts and interrogated distinct outcomes. Cox model analysis of 954 antiretroviral therapy-naïve HIV-1 seroconverters revealed a significant association between higher global TD and more rapid progression to AIDS as defined by the CDC in 1987 (AIDS-1987), after adjusting for race and HLA-B*57/27/35Px alleles (HR = 1.5 per one log_10_ increase in global TD score, *P* = 0.001; Table 2 and Fig. 3*A*), which are known to affect AIDS progression in this cohort (30). *HLA-B* TD provided the greatest contribution to the global effect, showing a significant association on its own (HR = 1.3, *P* = 0.008). Since there was no significant effect of TD on progression to CD4 cell counts <200/μL and AIDS-1993, definitions that precede an AIDS-defining illness (i.e., AIDS-1987), the detrimental effect of greater TD is likely to manifest later in the course of infection.

**Table 2.** Influence of *HLA class I* TD on progression to AIDS-1987 in seroconverters (*n* = 954) and HIV-1 viremia (*n* = 4.306)

	HR per log_10_ TD increase^†^	*P* value	VL est. per log_10_ TD increase^‡^	*P* value
*HLA-A/B/C*	1.5	0.001	0.17	3 × 10^−36^
*HLA-A*	1.2	0.2	0.10	8 × 10^−9^
*HLA-B*	1.3	0.008	0.06	4 × 10^−12^
*HLA-C*	1.1	0.6	0.10	2 × 10^−4^

^†^ Cox proportional hazards model adjusted for race and B*57, B*27, B*35-Px allelic effects.

^‡^ Longitudinal VL analysis adjusted for random effects due to each *HLA-A, HLA-B*, and *HLA-C* allele present in the cohort, the time after enrollment, and diploid HLA coding.

The influence of TD on viral load (VL) was validated in an independent cohort of 4,306 individuals for whom longitudinal VL measurements (HIV RNA copies/mL of blood) were available prior to initiation of antiretroviral therapy. Consistent with the Cox analysis, TD correlated positively with mean VL (mVL), where every 1 log_10_ increase in TD score associated with an increase of 0.21 mean log_10_ VL (*P* = 1.2 × 10^−6^; Fig. 3*B*). Analysis of longitudinal VL in the same set of patients indicated a 0.17 log_10_ increase in VL over time per 1 log_10_ increase in TD score (*P* = 3 × 10^−36^; Table 2), mirroring the results observed using mVL as an outcome. The *HLA-A* and *HLA-B* TD scores demonstrated similar effects when analyzed separately (Table 2).

## Discussion

*HLA class I* polymorphism defines multiple allele-specific features, including peptide specificity, mRNA/protein expression levels, and binding to innate immune receptors (31). Here, we characterize another property that differentiates HLA class I allotypes, their level of dependence on tapasin to bind and present peptides on the cell surface. Tapasin-independent allotypes have greater breadth in HIV peptide presentation to T cells, making it more difficult for the virus to adapt to these HLA types, thereby explaining the protective effect of tapasin-independent *HLA class I* genotypes (Fig. 3 and Table 1). We previously reported genetic epidemiological observations indicating an *HLA class I* heterozygote advantage against HIV (32), supporting a theory originally put forth by Doherty and Zinkernagel proposing that heterozygosity at class I results in a greater repertoire of viral peptides to which an individual can respond (33). These studies are further supported by recent theoretical data (34). Thus, there are at least two mechanisms by which *HLA class I* genotypes can enhance the breadth of the peptide binding repertoire and protect against viral disease: heterozygosity and tapasin independence.

Tapasin-dependent allotypes require the PLC for peptide loading, and only high affinity peptides are likely to dislodge tapasin from the peptide binding groove, allowing the HLA–peptide complex to proceed to the cell surface. Tapasin-independent allotypes do not require the PLC for peptide loading, resulting in greater promiscuity in the peptides they bind, including peptides that may often be of low affinity. In support of this model, B*44:02–peptide complexes were shown to be significantly more thermostable than B*44:05–peptide complexes, indicating that B*44:02, which strongly requires tapasin for peptide loading, assembles with higher affinity peptides than does B*44:05, an allotype that does not require tapasin for loading peptides (17).

Analogous to the protection against HIV conferred by tapasin-independent allotypes (i.e., those that bind a greater repertoire of peptides), greater promiscuity in peptide binding of chicken major histocompatibility complex (MHC) class I confers strong protection against Marek’s disease, a highly contagious disease in this species caused by an alphaherpes virus (35). Along the same lines, SIV vaccine studies in rhesus monkeys showed that successful protection required CTL responses to an exceptionally broad, low affinity peptide pool restricted by Mamu-E (36). Taken together, these studies in three distinct species point to greater breadth of the peptide binding repertoire as a protective host mechanism against viral disease. This may be of major consequence in successful vaccine design: Targeting epitopes restricted by the fastidious tapasin-dependent allotypes is likely critical for overall success of the vaccine, whereas the more promiscuous tapasin-independent allotypes may respond effectively to a broader range of vaccine antigens.

Apart from presenting a broader peptide repertoire, tapasin-independent allotypes would also be relatively beneficial if tapasin function is affected by the virus, as they would continue to present viral peptides to CTL. A recent HIV-host interactome study identified tapasin, among other host proteins, to associate with HIV-1 Env (37), raising the possibility that this interaction may impact peptide loading. In general, the ability to load peptides without being incorporated into the PLC, a characteristic of tapasin-independent alleles, would be beneficial if any component of the PLC, including tapasin, is inhibited under pathological conditions.

We previously reported that tapasin-independent genotypes associated with faster progression to death after HIV infection (14), which contrasts with our present findings. The previous study was premature in this regard, having clear limitations compared to that described herein: 1) smaller sample sizes (*n* = 496 vs. 954 for survival analyses) and no validation in an independent cohort (present study only, *n* = 4,306); 2) fewer alleles for which TD values were available (27 *HLA-B* vs. 97 *HLA-A/B/C* alleles); 3) inability to account for global TD levels since only *HLA-B* alleles were considered; 4) no functional explanation to support the genetic data. Overall, the increased power, reproducibility across cohorts, and functional data provided in the current work convincingly point to the protective effect of tapasin-independent *HLA class I* genotypes in HIV disease.

The impact of TD on outcome after HIV infection is clearly distinct from individual allelic effects, as the most protective allotype of all, HLA-B*57:01, is highly tapasin-dependent. B*57 allotypes are known to present multiple, highly conserved protective epitopes (30), an attribute that is rare among HLA allotypes. Rigorous peptide editing within the PLC of B*57 is likely to be advantageous, as it ensures that the high affinity, protective epitopes are loaded into the peptide binding groove and presented on the cell surface. This situation may be restricted to B*57 and perhaps a small set of other allotypes that present highly protective peptides. For the majority of HLA allotypes, however, enhanced breadth of the peptide repertoire conferred by tapasin independence favors immune control of HIV.

The range in breadth of the peptide presentation by distinct MHC allotypes described herein in humans mirrors that described in chicken, where “generalists” and “specialists” have been identified across class I allotypes (38). In humans, TD impacts this division (in a continuous manner), whereas in chicken, the structure of the peptide binding groove determines which category a specific allotype belongs, an example of convergent evolution. TD of specialists is likely to be protective against certain diseases, whereas tapasin independence of generalists protects against others. Thus, natural selection to maintain the broad distribution of TD at the population level contributes to the remarkable plasticity of the immune system in health and disease.

## Methods

### Human Subjects.

CTL responses were measured in a cohort of 989 individuals from Durban, South Africa (24). Longitudinal viral load and HLA class I data were available from a total of 5,114 HIV-1 infected individuals from six US cohorts and one European cohort: Adult Clinical Trials Group, AIDS Linked to i.v. Experience (ALIVE), US military HIV Natural History Study, Multicenter AIDS Cohort Study (MACS), Massachusetts General Hospital Controller Cohort, Study on the Consequences of Protease Inhibitor Era, and Swiss HIV cohort study. Seroconversion time and AIDS progression data were known for 1,112 patients from the District of Columbia gay cohort study, the Multicenter Hemophilia Cohort Study, the San Francisco City Clinic Cohort, MACS, and ALIVE. The representative institutional review boards approved this study. Each participant approved use of DNA samples for genetic analysis. All samples provided to us were deidentified.

### Cell Lines and Expression Constructs.

The human B cell lines .220 and .221 were generated previously by γ-radiation of 721 cells and do not express HLA-A/B and may express HLA-C/E/F (16, 39–42). These cell lines were grown in RPMI 1640 medium (Quality Biologicals) supplemented with 10% (vol/vol) fetal bovine serum (Atlanta Biologicals) and 1× Penicillin-Streptomycin-l-Glutamine Mixture (Lonza). HEK293T cells used for lentivirus production were maintained in advanced DMEM (Gibco) supplemented with 10% FBS and 2 mM l-glutamine (Gibco).

The HLA expression set included common alleles with frequencies >0.5% in European and African Americans, as well as the following alleles that are rare or absent in these populations: A*11:02, A*74:03, B*44:05, B*46:01, B*73:01, and C*18:02. Synthetically derived *HLA* allele fragments (LifeSct) were cloned into the modified pLVX-EF1α-IRES-Puro (Clontech) vector (43), in which EF1α was replaced with the SFFV promoter (pLVX-SFFV-IRES-Puro). The expression cassette encoded ZsGreen linked via self-cleaved P2A peptide to HLA with a FLAG-tag inserted between the allele-specific signal peptide and the mature protein. For tapasin expression, the vector was modified further by substitution of the puromycin resistance gene with the neomycin resistance gene (pLVX-SFFV-IRES-Neo). Tapasin was expressed with a V5-tag at the C terminus. Lentiviral transduction was performed as described in ref. 43. HLA-positive and tapasin-positive cells were selected using 0.25 μg/mL puromycin and 1 mg/mL G418 (Invivogen), respectively.

### Antibodies and Flow Cytometry.

Flow cytometric analyses were performed using the following monoclonal antibodies: APC anti-DYKDDDDK Tag (anti-FLAG; clone L5; BioLegend), HLA-ABC (W6/32) APC (eBioscience) (44), anti-V5-Tag Alexa Fluor 647 (clone SV5-Pk1; Bio-Rad). Cell surface staining of HLA expression was performed on cells grown in 24-well plates in 1 mL of volume on four different days, with 2-d intervals. For each experiment, 800 μL of cells (10^5^–10^6^) were used for staining, and 200 μL of cells were cultured further with fresh media. For tapasin detection, cells were fixed and permeabilized using the BD Cytofix/Cytoperm kit (Beckton Dickinson). Staining results were acquired using FACSCalibur flow cytometer (Beckton Dickinson), and analysis was performed using FlowJo software. Estimation of HLA expression levels involved several adjustments to the obtained APC MFI values: 1) background MFI of cells transduced with empty vector (i.e., no HLA insert) were subtracted; 2) MFI values were normalized to the average MFI across samples to adjust for daily instrumental variation; 3) MFI values were normalized to the level of ZsGreen to adjust for the level of transduction; 4) the numerator in the calculation of TD for each allele was the average expression levels of the corresponding allele in .220tpn cells expressing the R240 and T240 tapasin variants.

### HLA Genotyping and TD Assignment.

*HLA* typing in the HIV-1 cohorts was performed using a targeted next generation sequencing method. Briefly, locus-specific primers are used to amplify polymorphic exons of *HLA-A, HLA-B, HLA-C* genes with Fluidigm Access Array (Fluidigm). The Fluidigm PCR amplicons were pooled and subjected to sequencing either on the Roche 454 platform (Roche) or the Illumina MiSeq platform (Illumina). *HLA* alleles and genotypes were called using the Omixon HLA Explore (beta version) software (Omixon). TD values determined for each HLA allotype are shown in Fig. 1 and *SI Appendix*, Table S1. Genotypic TD at each locus for each individual were calculated by summing the TD values of the two alleles: TD(*HLA-A*) = TD(*A1*) + TD(*A2*); TD(*HLA-B*) = TD(*B1*) + TD(*B2*); TD(*HLA-C*) = TD(*C1*) + TD(*C2*), where *A1*/*A2*, *B1*/*B2*, and *C1*/*C2* represent the two alleles at *HLA-A*, *HLA-B*, and *HLA-C*, respectively. The global TD value for each individual was calculated by summing the TD scores at the three loci: TD(*HLA-A/B/C*) = TD(*HLA-A*) + TD(*HLA-B*) + TD(*HLA-C*). We were able to assign TD to 84% (4,306/5,114) and 86% (954/1,112) of individuals in the longitudinal VL and seroconverter cohorts, respectively. In the African cohort (ELISpot data), complete four-digit *HLA-A/B/C* genotypes were available for 853 individuals, of which TD scores were assigned to 638 (75%).

### HLA Class l Epitope Prediction.

Epitope predictions for HIV-1 was performed by using the SMM (26) and NetMHCpan EL 4.0 (27) algorithms available in the T cell section of Immune Epitope Database analysis tools (www.IEDB.org). In both cases, predicted affinity values (IC_50_ nanomolar) were retrieved for the specific *HLA* allele when applicable based on the selected algorithm. HIV-1 clade C peptides consisted of overlapping 18-mers as previously described (24). For prediction purposes, for each 18-mer, all of the possible 9-mers and 10-mers were derived and the lowest IC_50_ was assigned. The number of epitopes per HLA allele was calculated for both algorithms based on the number of peptides predicted to have an IC_50_ ≤ 500 nM.

### Statistics.

To identify HLA-peptide associations in the HIV-1 ELISpot assay (Fig. 2*A*), we used Fisher’s exact test (forward selection) to identify significant (*q* < 0.05) associations between the recognition of individual peptides and the expression of particular HLA class I alleles. All HLA class I alleles expressed at a phenotypic frequency of >3% were included in the analysis.

For disease association, regression analyses were performed in R (v 3.6.0) using the following packages: lme4 and ggplot2. Analyses of VL was performed using the lmer function. Random effects due to each *HLA-A*, *HLA-B*, and *HLA-C* allele, the time postenrolment, and a correction for diploid *HLA* allele coding were included in the models. We tested two alternative outcomes: a geometric mean HIV VL (mVL) at all measurement timepoints (mean log_10_VL) and the log_10_ transformed HIV VL at each timepoint. For survival analyses, we used the coxph function (survival package) and estimated the time to AIDS (CDC 1987 and 1993 definition), CD4+ T cell count <200 cells/μL, and death.

Correlation tests were performed using GraphPad Prism.

## Supplementary Material

Supplementary File

## Data Availability

All experimental results described in the paper are presented in the table and figures. All clinical data underlying the study are available from the cohorts upon application.
