# Supplementary material for: HLA tapasin independence: broader peptide repertoire and HIV control
Source: Proc Natl Acad Sci U S A. 2020 Oct 23;117(45):28232–8. doi: 10.1073/pnas.2013554117 (PMC7668082; doi:10.1073/pnas.2013554117)
Supplement: Supplementary File [file pnas.2013554117.sapp.pdf]

PNAS
www.pnas.org

Supplementary Information for

HLA tapasin independence: broader peptide repertoire and HIV control

Arman A. Bashirova, Mathias Viard, Vivek Naranbhai, Alba Grifoni, Wilfredo Garcia-Beltran, Marjan Akdag, Yuko Yuki, Xiaojiang Gao, Colm O'hUigin, Malini Raghavan, Steven Wolinsky, Jay H. Bream, Priya Duggal, Jeremy Martinson, Nelson L. Michael, Gregory D. Kirk, Susan P. Buchbinder, David Haas, James J. Goedert, Steven G. Deeks, Jacques Fellay, Bruce Walker, Philip Goulder, Peter Cresswell, Tim Elliott, Alessandro Sette, Jonathan Carlson, Mary Carrington.
Paste the full author list here

Mary Carrington
Email: carringm@mail.nih.gov

This PDF file includes:

Supplementary text
Figures S1 to S4
Table S1

www.pnas.org/cgi/doi/10.1073/pnas.2013554117

Supplementary Information Text

Extended Acknowledgements

Data in this manuscript were collected in part by the Adult Clinical Trials Group (ACTG) supported by an ACTG Network Leadership Grant Award. We gratefully acknowledge the SDMC, participating CRSs, and Specialty Laboratories of the ACTG. Research reported in this publication was supported by the National Institute of Allergy and Infectious Diseases of the National Institutes of Health under Award Number UM1 AI068634, UM1 AI068636 and UM1 AI106701. The content is solely the responsibility of the authors and does not necessarily represent the official views of the National Institutes of Health.

Data in this manuscript were collected in part by the MACS/WIHS Combined Cohort Study (MWCCS). The contents of this publication are solely the responsibility of the authors and do not represent the official views of the National Institutes of Health (NIH). MWCCS (Principal Investigators): Atlanta CRS (Ighovwerha Ofotokun, Anandi Sheth, and Gina Wingood), U01-HL146241-01; Baltimore CRS (Todd Brown and Joseph Margolick), U01-HL146201-01; Bronx CRS (Kathryn Anastos and Anjali Sharma), U01-HL146204-01; Brooklyn CRS (Deborah Gustafson and Tracey Wilson), U01-HL146202-01; Data Analysis and Coordination Center (Gypsyamber D'Souza, Stephen Gange and Elizabeth Golub), U01- HL146193-01; Chicago-Cook County CRS (Mardge Cohen and Audrey French), U01-HL146245-01; Chicago-Northwestern CRS (Steven Wolinsky), U01-HL146240-01; Connie Wofsy Women's HIV Study, Northern California CRS (Bradley Aouizerat and Phyllis Tien), U01-HL146242-01; Los Angeles CRS (Roger

Detels and Otoniel Martinez-Maza), U01-HL146333-01; Metropolitan Washington CRS (Seble Kassaye and Daniel Merenstein), U01-HL146205-01; Miami CRS (Maria Alcaide, Margaret Fischl, and Deborah Jones), U01-HL146203-01; Pittsburgh CRS (Jeremy Martinson and Charles Rinaldo), U01- HL146208-01; UAB-MS CRS (Mirjam-Colette Kempf and Deborah Konkle-Parker), U01-HL146192-01; UNC CRS (Adaora Adimora), U01-HL146194-01. The MWCCS is funded primarily by the National Heart, Lung, and Blood Institute (NHLBI), with additional co-funding from the Eunice Kennedy Shriver National Institute Of Child Health & Human Development (NICHD), National Human Genome Research Institute (NHGRI), National Institute On Aging (NIA), National Institute Of Dental & Craniofacial Research (NIDCR), National Institute Of Allergy And Infectious Diseases (NIAID), National Institute Of Neurological Disorders And Stroke (NINDS), National Institute Of Mental Health (NIMH), National Institute On Drug Abuse (NIDA), National Institute Of Nursing Research (NINR), National Cancer Institute (NCI), National Institute on Alcohol Abuse and Alcoholism (NIAAA), National Institute on Deafness and Other Communication Disorders (NIDCD), National Institute of Diabetes and Digestive and Kidney Diseases (NIDDK). MWCCS data collection is also supported by UL1- TR000004 (UCSF CTSA), P30-AI-050409 (Atlanta CFAR), P30-AI-050410 (UNC CFAR), and P30-AI- 027767 (UAB CFAR).

The Swiss HIV Cohort Study is supported by the Swiss National Science Foundation (grant #177499) and by the SHCS research foundation . The data are gathered by the Five Swiss University Hospitals, two Cantonal Hospitals, 15 affiliated hospitals and 36 private physicians (listed in http://www.shcs.ch/180-health-care-providers). Members of

the Swiss HIV Cohort Study: Aebi-Popp K, Anagnostopoulos A, Battegay M, Bernasconi E, Böni J, Braun DL, Bucher HC, Calmy A, Cavassini M, Ciuffi A, Dollenmaier G, Egger M, Elzi L, Fehr J, Fellay J, Furrer H, Fux CA, Günthard HF (President of the SHCS), Haerry D (deputy of "Positive Council"), Hasse B, Hirsch HH, Hoffmann M, Hösli I, Huber M, Kahlert CR (Chairman of the Mother & Child Substudy), Kaiser L, Keiser O, Klimkait T, Kouyos RD, Kovari H, Ledergerber B, Martinetti G, Martinez de Tejada B, Marzolini C, Metzner KJ, Müller N, Nicca D, Paioni P, Pantaleo G, Perreau M, Rauch A (Chairman of the Scientific Board), Rudin C, Scherrer AU (Head of Data Centre), Schmid P, Speck R, Stöckle M (Chairman of the Clinical and Laboratory Committee), Tarr P, Trkola A, Vernazza P, Wandeler G, Weber R, Yerly S.

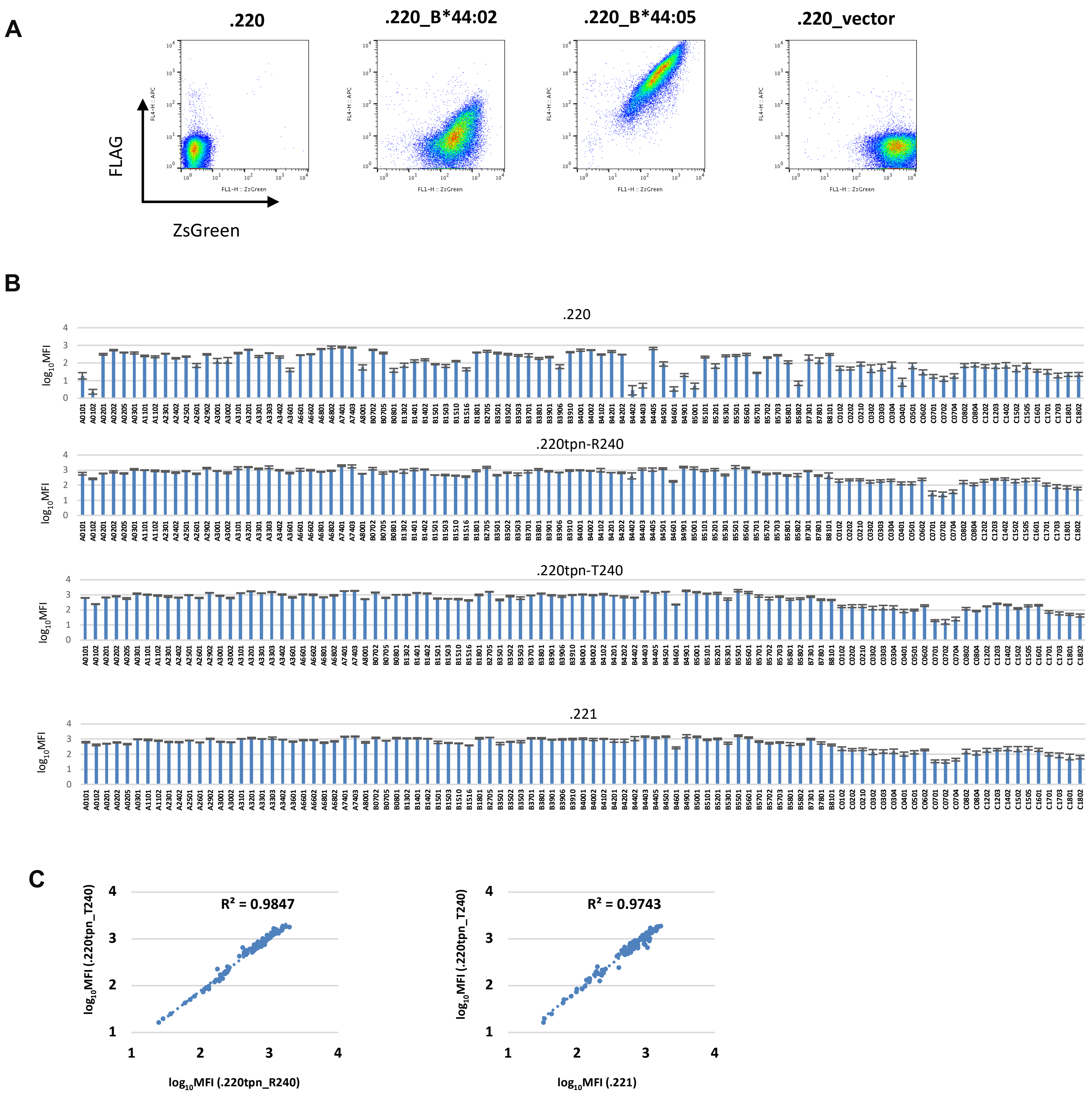

Fig. S1. Flow cytometric analysis of HLA expression in the presence or absence of tapasin. (A) Representative flow cytometry plots show ZsGreen fluorescence and anti-FLAG mAb binding to parental .220 cells, and .220 cells transduced with *HLA-B*44:02*, *HLA-B*44:05*, or “empty vector” (no HLA) constructs. ZsGreen fluorescence marks cells that were lentivirally transduced. Tapasin-dependent HLA-B*44:02 is expressed at lower levels compared to tapasin-independent HLA-B*44:05. (B) HLA class I allotype expression levels in .220, .220tpn-R240, .220tpn-T240, and .221 cells. The MFI values and standard deviations represent data obtained from four measurements performed on different days on the same cells maintained in culture. (C) Expression levels measured in .220tpn_T240 and .220tpn_R240 cells (left), as well as in .220tpn_T240 and .221 cells (right) demonstrate significant correlations, respectively.

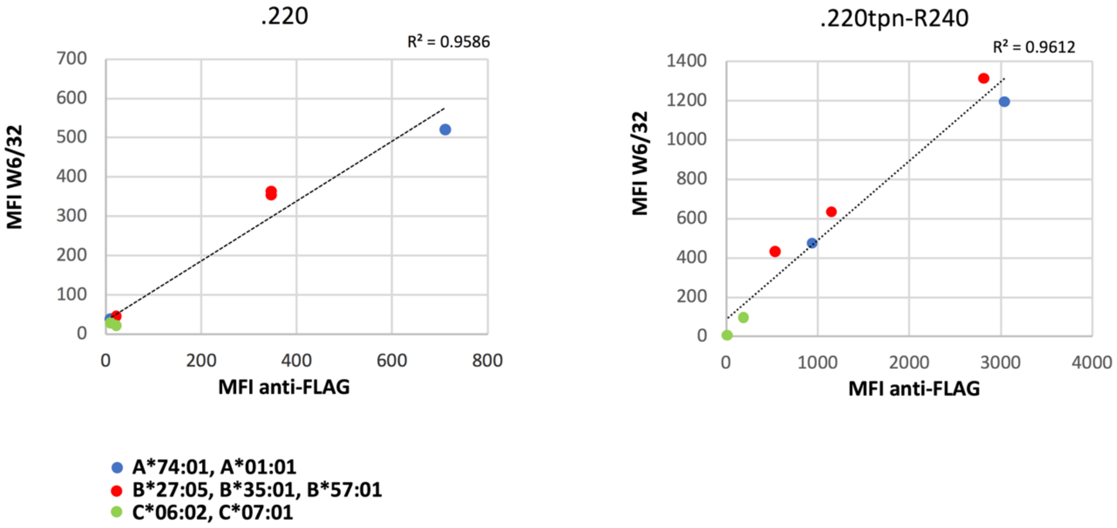

Fig. S2. HLA expression levels measured using W6/32 mAb and anti-FLAG mAb strongly correlate. Expression of two HLA-A, three HLA-B and two HLA-C allotypes were analyzed by flow cytometry on the surface of .220 (left) and .220tpn-R240 (right) cells.

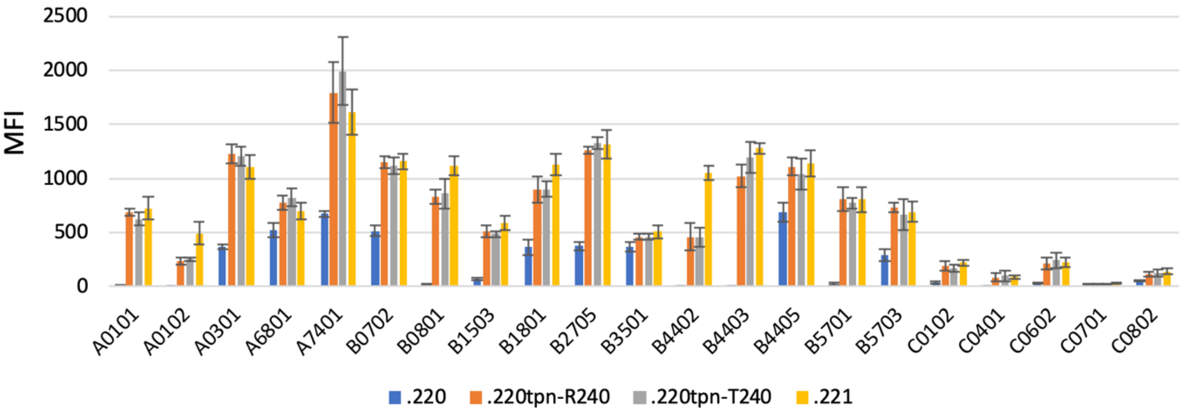

Fig.S3. Comparison of HLA allotype expression levels across .220, .220tpn, and .221 cells. Tapasin-negative and -positive cells transduced with a subset of *HLA* alleles were analyzed by flow cytometry using anti-FLAG mAb on the same day. Expression levels in tapasin-positive cells are similar across most allotypes, although A*01:02, B*44:02, and, to a lesser extent, B*08:01 and C*07:01 showed lower expression on .220tpn than on .221 cells ($p=0.03$, Wilcoxon test). These differences may reflect some level of insufficiency of the exogenous compared to endogenous tapasin for assembly of these particular allotypes. There was no difference in expression level of each allele on .220tpn cells transduced with either R240 or T240 tapasin ($p>0.05$, Wilcoxon test). The MFI values and standard deviations represent data obtained from four measurements performed on different days using the same cells maintained in culture.

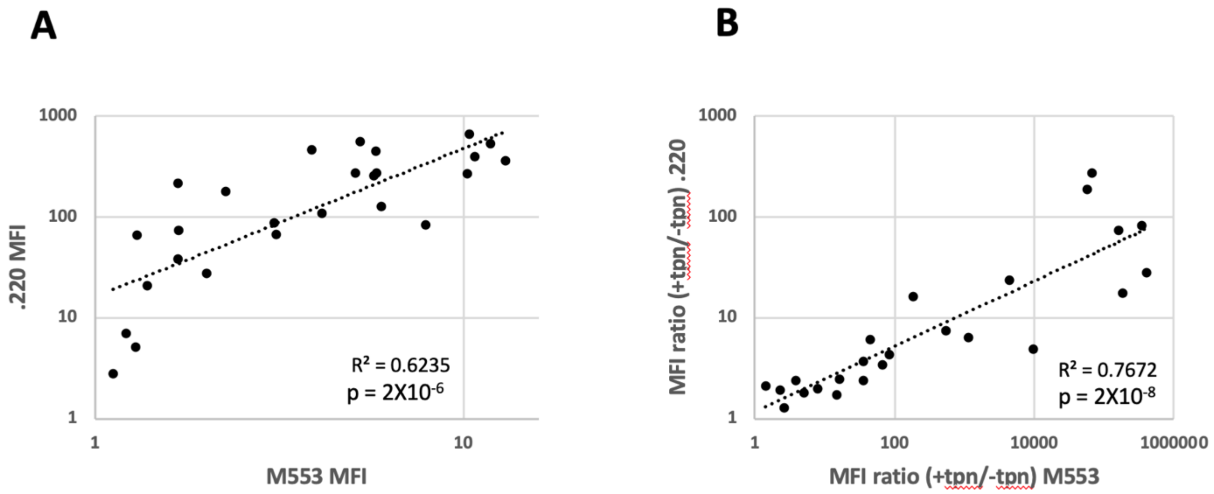

Fig. S4. Correlation between HLA-B expression on .220 and M553 cells (M553 data derived from Rizvi et al. (14). (A) Comparison of HLA-B expression levels in .220 vs. M553 cells. (B) Comparison of HLA-B MFI ratios (+tpn/-tpn) in .220 vs. M553 cells. Each dot represents an HLA-B allotype that was evaluated for TD in both the present study and Rizvi et al.

Table S1. TD of HLA class I allotypes defined as the ratio of cell surface expression levels in .220tpn over that in .220 cells.

HLA	MFI ratio (+tpn/-tpn)	SD	HLA	MFI ratio (+tpn/-tpn)	SD
A*01:01	32.11	13.77	B*40:02	1.71	0.11
A*01:02	109.86	35.04	B*41:02	3.45	0.53
A*02:01	2.02	0.22	B*42:01	1.72	0.21
A*02:02	1.45	0.16	B*42:02	2.31	0.21
A*02:05	1.49	0.12	B*44:02	190.40	121.20
A*03:01	3.14	0.46	B*44:03	274.16	85.74
A*11:01	4.00	0.40	B*44:05	1.82	0.33
A*11:02	3.99	0.57	B*45:01	16.31	4.70
A*23:01	2.36	0.20	B*46:01	60.66	17.61
A*24:02	3.61	0.35	B*49:01	82.40	16.89
A*25:01	3.91	0.29	B*50:01	286.17	110.15
A*26:01	8.33	2.14	B*51:01	4.92	0.66
A*29:02	4.38	0.45	B*52:01	17.51	5.42
A*30:01	6.52	2.01	B*53:01	1.96	0.31
A*30:02	4.54	1.71	B*55:01	6.46	1.15
A*31:01	3.67	0.50	B*56:01	4.60	0.73
A*32:01	2.92	0.20	B*57:01	28.43	3.42
A*33:01	5.34	0.71	B*57:02	2.74	0.41
A*33:03	4.20	0.48	B*57:03	2.46	0.24
A*34:02	4.66	0.67	B*58:01	4.30	0.86
A*36:01	16.33	3.80	B*58:02	73.28	22.75
A*66:01	3.77	0.42	B*73:01	3.97	1.38
A*66:02	3.16	0.27	B*78:01	3.33	1.23
A*68:01	1.17	0.09	B*81:01	1.45	0.34
A*68:02	1.19	0.21	C*01:02	3.67	1.09
A*74:01	2.31	0.28	C*02:02	4.17	0.95
A*74:03	2.40	0.31	C*02:10	2.34	0.67
A*80:01	9.70	3.34	C*03:02	3.19	1.65
B*07:02	2.37	0.28	C*03:03	3.01	1.42
B*07:05	1.72	0.24	C*03:04	2.32	1.01
B*08:01	23.78	5.94	C*04:01	13.59	7.47
B*13:02	12.35	3.68	C*05:01	1.68	0.66
B*14:01	9.48	1.76	C*06:02	7.43	2.52
B*14:02	7.68	1.01	C*07:01	1.38	0.45
B*15:01	6.10	0.49	C*07:02	1.63	0.66
B*15:03	7.52	1.49	C*07:04	1.69	0.59
B*15:10	3.72	0.36	C*08:02	1.97	0.57
B*15:16	9.18	1.82	C*08:04	1.28	0.36
B*18:01	2.40	0.23	C*12:02	2.83	0.69
B*27:05	3.38	0.47	C*12:03	3.76	1.17
B*35:01	1.26	0.16	C*14:02	3.19	1.19
B*35:02	2.38	0.29	C*15:02	3.31	1.39
B*35:03	2.10	0.36	C*15:05	2.99	1.17
B*37:01	3.13	0.72	C*16:01	5.98	1.33
B*38:01	6.39	0.84	C*17:01	2.92	0.98
B*39:01	4.10	0.41	C*17:03	3.63	1.29
B*39:06	11.95	3.11	C*18:01	2.76	0.80
B*39:10	2.32	0.21	C*18:02	2.28	0.66
B*40:01	1.90	0.28			
